# Endless Urban Growth? On the Mismatch of Population, Household and Urban Land Area Growth and Its Effects on the Urban Debate

**DOI:** 10.1371/journal.pone.0066531

**Published:** 2013-06-20

**Authors:** Dagmar Haase, Nadja Kabisch, Annegret Haase

**Affiliations:** 1 Humboldt Universität zu Berlin, Department of Geography, Helmholtz Centre for Environmental Research – UFZ, Leipzig, Germany; 2 Helmholtz Centre for Environmental Research – UFZ, Leipzig, Germany; University of Florida, United States of America

## Abstract

In European cities, the rate of population growth has declined significantly, while the number of households has increased. This increase in the number of households is associated with an increase in space for housing. To date, the effects of both a declining population and decreasing household numbers remain unclear. In this paper, we analyse the relationship between population and household number development in 188 European cities from 1990–2000 and 2000–2006 to the growth of urban land area and per capita living space. Our results support a trend toward decreasing population with simultaneously increasing household number. However, we also found cites facing both a declining population and a decreasing household number. Nevertheless, the urban land area of these “double-declining” cities has continued to spread because the increasing per capita living space counteracts a reduction in land consumption. We conclude that neither a decline in population nor in household number “automatically” solve the global problem of land consumption.

## Introduction and Setting the Scene

Population, which directly influences the consumption of goods, is one of the most important drivers of global environmental change [1], [2]. Processes related to demographic changes also have significant impacts on urbanisation and the growth of cities. At the current moment, in Europe, despite the decreased rate of population growth [3], [4], [5], [6], [7], [8], the scientific literature reports a significant increase in the number of households due to the trend toward smaller households, primarily in cities [9], [10], [11]. The growth of household numbers is associated with an increase in land consumption [12] due to the additional demand for land area to accommodate new housing. This land area growth leads to an increase in impervious surfaces [13], [14], [5 for the EU].

Liu et al. using a highly selective and extremely diverse sample of case studies that included New Zealand, Italy, Brazil, Indian River County in the US, Mauritius and China, argue that the increased number of households influences the per capita consumption of land [12]. These researchers determined that the global growth in household numbers was more rapid than the total population growth between 1985 and 2000. Furthermore, these authors identified that even when the total population size was declining, the number of households was substantially increasing.

However, neither the dynamics in household number in cites with an associated decline in population nor the effects of decreased household numbers on land area growth in cities have been included in research to date. When household numbers are declining, too, one could assume that this decline should cause a declining demand for new living spaces and thus a reduction in urban land consumption, which could be one solution to the global problem of ongoing land consumption. Because in housing markets, it is households and not individuals who are key players deciding for or against a “living space”, i.e., a flat or house, the effects of household number change are crucial for assessing future land consumption trends for urban areas. Such analyses in a continental scale that include a number of cities and do not rely on specific sample cases is currently lacking in the literature.

Liu et al. convincingly demonstrated that population decline as such does not lead to reduced land consumption under the condition of further growth in household numbers [12]. However, there is still a gap in the current knowledge with respect to what happens if household numbers also decrease. Further, there is limited information concerning the effects of a “double” decrease (i.e., of population and household numbers) on land area growth. Could we assume that a decrease in household numbers would have a positive effect on land area use, thereby leading to a reduction in further land consumption? To tackle this issue, we investigate whether double downward development of the two explanatory variables, total population number and number of households, supports a reduction in land consumption on a continental scale.

Set against this background, in our paper, we aim to answer the following two questions:

1. Does the effect of a double downward development of population and household number decline lead to a reduction in urban land area?
2. If not, what might be the drivers for on-going urban land consumption?

## Materials and Methods

To answer both questions, we analysed the development of population and household number in 188 European cities participating in the Urban Audit data collection between 1990, 2000 and 2006 (cities and their population numbers are listed in Table S1). In addition, we measured the annual growth rates of urban land area and per capita living space for these periods in time.

Data on population number, household number, including number of one-person households, per capita living space (m^2^/inhabitant) and urban land area were extracted from various publicly available statistical databases (Table 1): the Urban Audit database [15] and the European Commission’s Corine Land Cover Programme [16]. Urban land area (in ha) is defined as the aggregated value of the continuous and discontinuous urban fabric in the Corine Land cover data set. Continuous urban fabric includes buildings, approach road networks, and artificially surfaced areas (e.g., parking lots) with coverage of more than 80% of the total surface (of a polygon with minimum mapping size of 25 ha) [17]. Accordingly, discontinuous urban fabric refers to similar areas but is associated with vegetated areas and bare soil, where between 30 to 80% of the total surface is impermeable.

**Table 1:** Data, temporal scale and sources.

Data	Calculated variable/period	Data source and temporal scale
Administrative boundaries	–	Urban Audit database^1^ 2004
Demography: Population number, number ofhousehold, number of one-person households	Annual growth rate 1990–2000 and 2000–2006	Urban Audit database^1^1991, 2001, 2004^2^
Urban land area: aggregation of CORINE classes 111 (continuous urban fabric) and 112 (discontinuousurban fabric)	Annual growth rate 1990–2000 and 2000–2006	Corine Land Cover EEA^3^1990, 2000, 2006
Per capita living space (m^2^/inhabitant)	Annual growth rate 1990–2000 and 2000–2006	Urban Audit database^1^1991, 2001, 2004^2^

1 www.urbanaudit.org.

2 The urban audit data collection period started in 2004 but for some cities data refer to 2005 or 2006. This was considered in the calculation of the growth rates.

3 European Environmental Agency, CORINE Land Cover Programme http://www.eea.europa.eu/themes/landuse/clc-download, last visited 06 January 2010.

The three time periods of 1990, 2000 and 2006 and the respective time intervals were chosen as they correspond to the Corine Land Cover data, which provide land use information for the same points in time for all of Europe. The list of the selected cities included in our analysis represents those relevant datasets available in the Urban Audit database. Overall, 327 cities participated in the Urban Audit, which represents a data collection of comparable statistics and indicators for European cities every three years. Unfortunately, data are not provided for each city for every period. Thus, a city was deleted from our sample in the case of identified ambiguities between time periods. Finally, a sample of 188 cities for the period 1990–2000 and 118 cities for the period 2000–2006 represent the foundation for our study.

The mean annual growth rates for all cities were computed for each variable *x* as Δ*px* and as *HH* for the number of households. The calculations represent simple percentage changes in the values from one point in time to the second point in time and subsequently divided by the respective time span (e.g., 10 years for 1990–2000) to determine the annual changes:
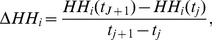
(1)where *HH_i_* are the number of HH in one city *i* in the year t_j_ with j  = 1,., 3 and 1 = 1990; 2 = 2000 and 3 = 2006 and
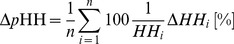
(2)with n = 188 and 118 and x =  number of households (HH).

The mean annual growth rates of population number (Pop), per capita living space (LSp), and urban land area (U) were calculated accordingly.

In the calculation of the mean annual growth rates, data were trimmed at the highest 5% values of each variable to attenuate the influence of outliers [18]). In the case of the variable of urban land area, we additionally calculated a weighted mean value of the annual changes, where the averaging was weighted by city size. We used this weighting to reduce the possible influence of city size, as urban land area changes in a smaller city have more influence on the value of the mean than an identical-sized change in a larger city; for more details on the calculation of the weighted mean see [19].

**Figure 1:**
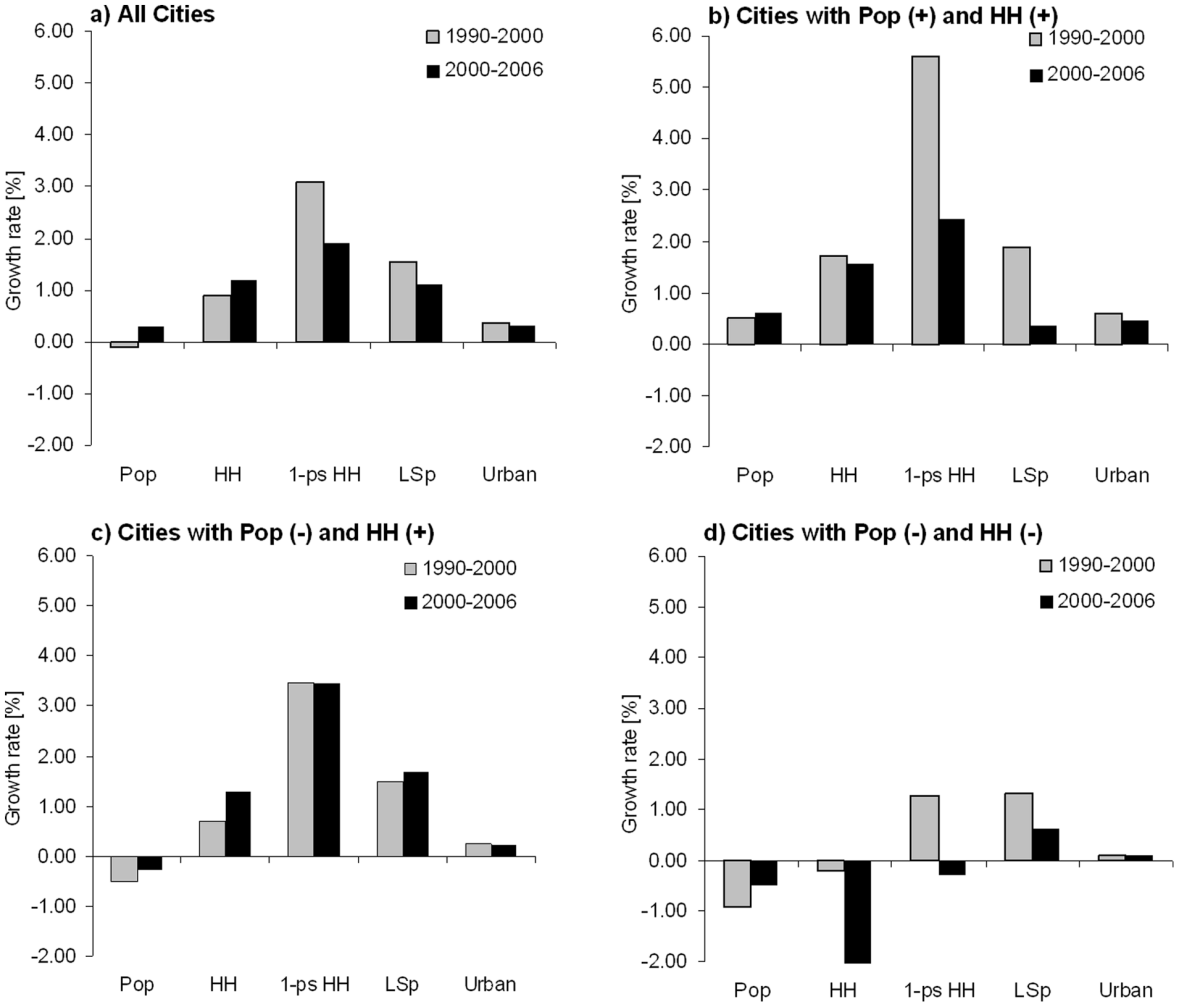
Mean annual growth rates of the aggregate population size (Pop), the number (no) of households (HH), the per capita living space (LSp) and urban land area (U) in 188 (118) European cities 1990–2000 and 2000–2006.

Mean values for all variables were calculated for different sub-samples, such as only for those cities with a declining/growing population or for those cities with growing/declining household numbers. The mean values are shown as bar charts in Figure 1. To depict the spatial distribution of cities, their population and household number development within Europe, maps were produced using the Geographical Information System of ArcGIS 10.0.

## Results

**Figure 2:**
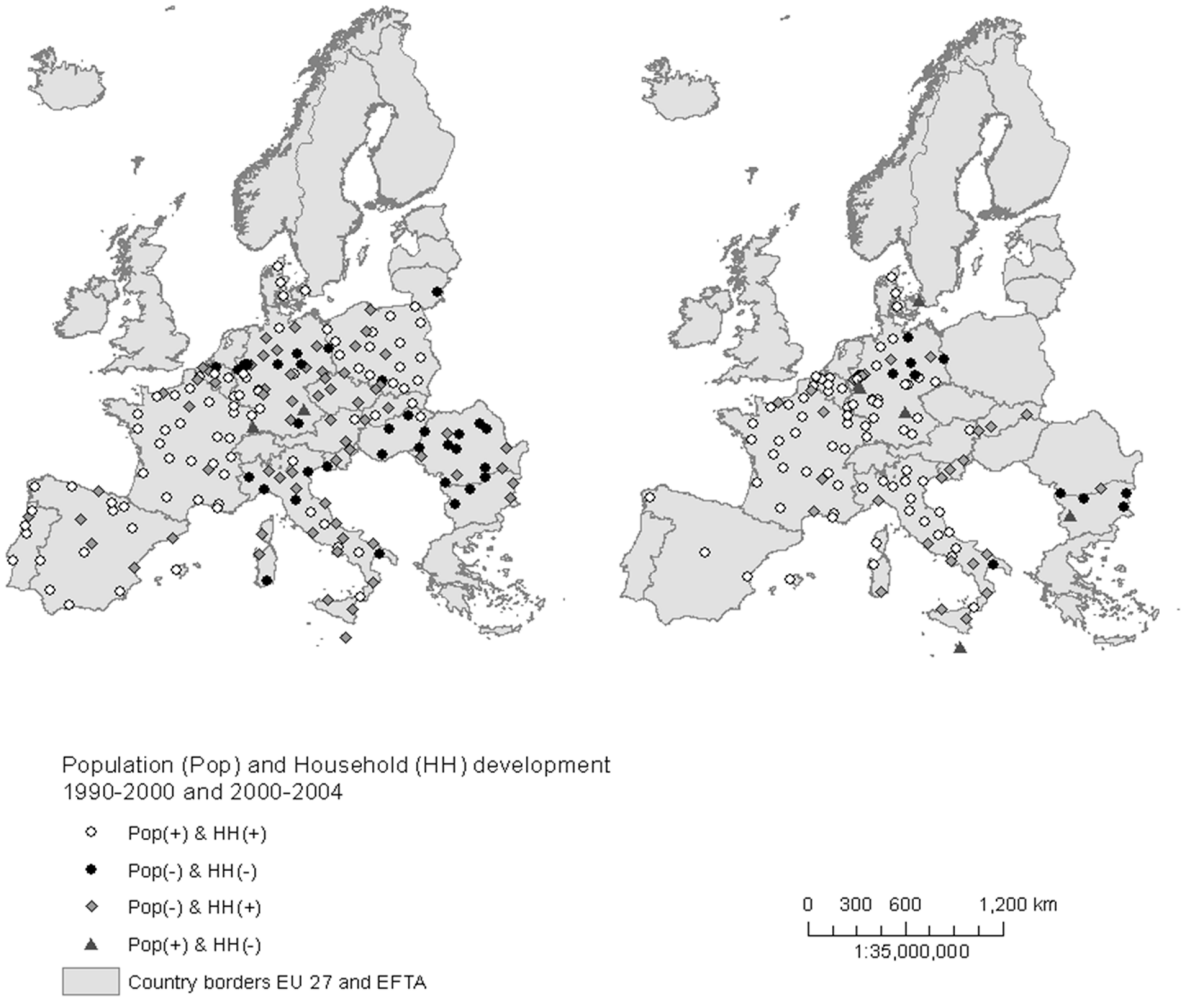
Distribution of cities in Europe according to population and household development in 1990–2000 and 2000–2004. POP – Population, HH – Households,+positive growth rate, −negative growth rate. Note: No data on household number was available for the period 2000–2004 for Poland, the Czech Republic, Romania, Switzerland, Hungary, Lithuania and Portugal.

Table 2 summarises the number of cities and their percentage share in four different categories. These data include a category with only those cities with positive growth rates for both population and household number: Pop (+) and HH (+); a second category with those cities showing only negative values for population and household number: Pop (−) and HH (−); and finally two categories where either population number or household number was positive, while the other variable was negative: Pop (−) and HH (+) and Pop (+) and HH (−). These categories are also used in Figure 1 where the trimmed mean values are shown as bar charts and in the maps in Figure 2.

**Table 2:** Comparison between growth rates of the population (Pop) and household numbers (HH) in European cities for the periods of 1990–2000 (n = 188) and 2000–2006 (n = 118).

Relationship between Pop and HH	European cities			
	1990–2000		2000–2006	
	n	%	n	%
Pop(+) and HH(+)	82	43.6	74	62.7
Pop(+) and HH(−)	[2] *	[1.1]*	[6] *	[5.1]*
Pop(−) and HH(+)	68	36.2	27	22.9
Pop(−) and HH(−)	36	19.1	11	9.3
All cities	188	100.0	118	100.0

Abbreviations: n = number of cases; Pop = Population, HH = number of households;+positive growth rate, − negative growth rate.

* numbers too small for statistical analysis, thus not included in further figures.

### Population and Household Number Development in European Cities Since 1990

In our analysis, we identified 82 European cities for 1990–2000 and 74 cities for 2000–2006, which exhibited simultaneously increasing population and household numbers. For all cases, the growth rates of the household number were significantly higher than the growth rates of the population (Fig. 1a and 1b). These growing cities are primarily situated in Western and Southern European countries, such as in Denmark, France or Spain and Italy, particularly since 2000. However, a number of Polish and Slovak cities in Eastern Europe also exhibited both population and household number growth (at least for the period of 1990–2000). Conversely, in a number of cities, household numbers are still growing approximately 1% per year, while more than one third of the sample (36%) shows a decline in population numbers between 1990 and 2000, and nearly 20% between 2000 and 2006 (Fig. 1c). The maps in Figure 2 indicate that those cities can be found in Central and Western European countries, such as Germany, Austria and Belgium, but also in Italy and Eastern Europe, as in Poland and the Czech Republic. We also identified 36 cities that faced a decline in household numbers between 1990 and 2000 (decline −0.21%), and 11 cities between 2000 and 2006 (decline −1.78) accompanied by a population decline (Fig. 1d). These declining cities are situated in the eastern part of Germany and in Eastern Europe, as well as in western Germany, Italy and Belgium, for both time periods.

Figure 1 also shows the growth rates for the number of one-person households. In nearly all investigated cities, their growth rate is highly positive, regardless of whether the population or household number increases or decreases. There is, however, one exception, which is as follows: Fig. 1d presents that the growth rate for the number of one-person households is negative in the case of European cities with a declining population and declining household numbers for the period of 2000–2006, while it was positive for the period 1990–2000.

### Development of Urban Land Area and Living Space in Declining and Growing Cities

Figure 1 further shows the growth rates for the per capita living spaces in European cities. We identified an overall annual increase of approximately 1.5% for all cities in both periods (Fig. 1a). Particularly evident, however, is the mismatch in those cities with a declining population and declining household numbers (Fig. 1d). In these cities, the per capita living space increases by 1.3% per year in the first period and 0.6% in the second period, while annual growth rates of population and household numbers are negative. With respect to urban land area, all growth rates were positive, although on a low level (values between 0.1 and 0.6) in both reported periods of time. The growth rates of urban land area are even positive in cities with a declining population and declining household numbers (Fig. 1d). Thus, urban land area continuously increases regardless of growth or decline in population or household number.

**Figure 3:**
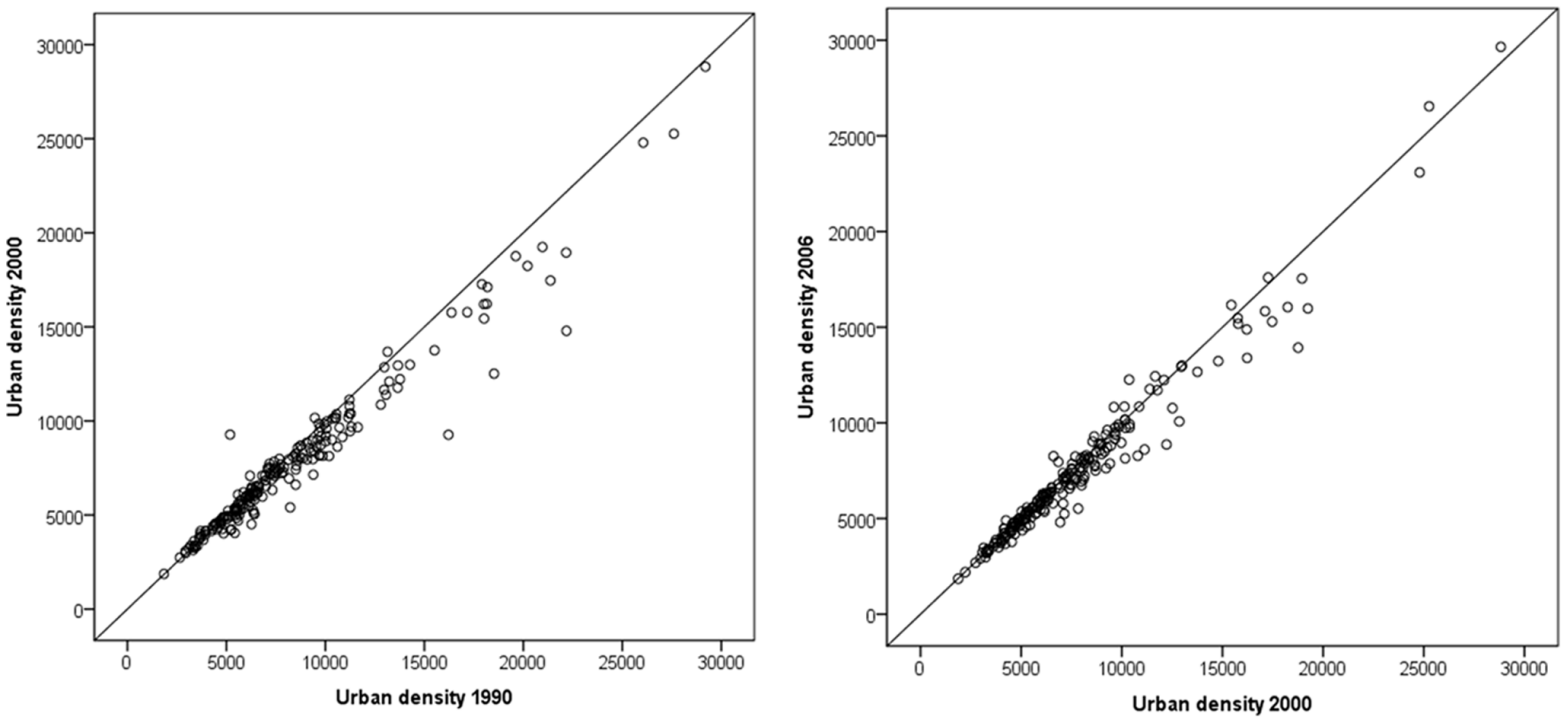
Population densities of European cities represented for 1990 compared to 2000 and for 2000 compared to 2004. Cities on the right side of the dividing line show a decline in density values compare to the other point in time. Note: Population density is shown as built-up density which is the population number per km^2^ urban area.

Urban density was at least to some extent already included in the analysis because we looked at growth of urban area and population number. Urban density was defined as inhabitants per urban (built-up) area. To show a possible change in urban density, Figure 3, reporting on the change of urban density for the two time periods, shows that density rather declines for a number of cities in the first period while there is nearly no change compared to the second period.

## Discussion

### Uncovering Household Number Dynamics as a Driver of Urban Land Area Growth

In our analysis, we identified European cities with declining household numbers and declining population number in Eastern European countries, as well as in Germany and Italy. The decline in household numbers may be the result of considerable population losses in cities, in which the losses cannot be balanced by the general growth in household numbers, a situation which can currently be found most frequently in post-socialist cities across Eastern Europe [20], [21], [22]. In a sample of more than 300 European cities, Turok and Mykhnenko showed that most of the Eastern European cities had been facing population declines since the 1990s, while the population numbers continued to increase in Western European cities, although on lower levels. Similarly, Kabisch and Haase identified a severe decline in population numbers in Eastern European cities, which was highest in the period 1990–2000 [10]. However, none of these studies focussed on parallel (declining) household development.

Our results also identified a number of European cities with increasing household numbers regardless population growth or decline. This growth is primarily attributed to the reduction in average household size, which has also accelerated worldwide in the last decades [13]. Several comparative investigations and a number of illustrative European case studies [21], [23], [24] have attempted to find explanations for this widespread European phenomenon. One explanation is that the increasing household numbers are a result of demographic change and, more precisely, of shifts summarised by the term second demographic transition [25]. These shifts have led to a decrease in mean household size and an increase in small and smallest households of <2 persons, which is most prominent in large cities, reaching rates of even >50 per cent for all households [26].

### The Role of One-person Households

As Figure 1 shows, the number of one-person households grew from 1990 to 2000, even in the case of cities with a decline in population and household number; however, in the second time period from 2000 to 2006, this finding was not noted. The result of a decline in one-person households accompanied by a decline in population and household numbers in the second period might have been caused by a shorter time period, missing data or a small sample size.

### The Invisible Variable: per Capita Living Space

**Figure 4:**
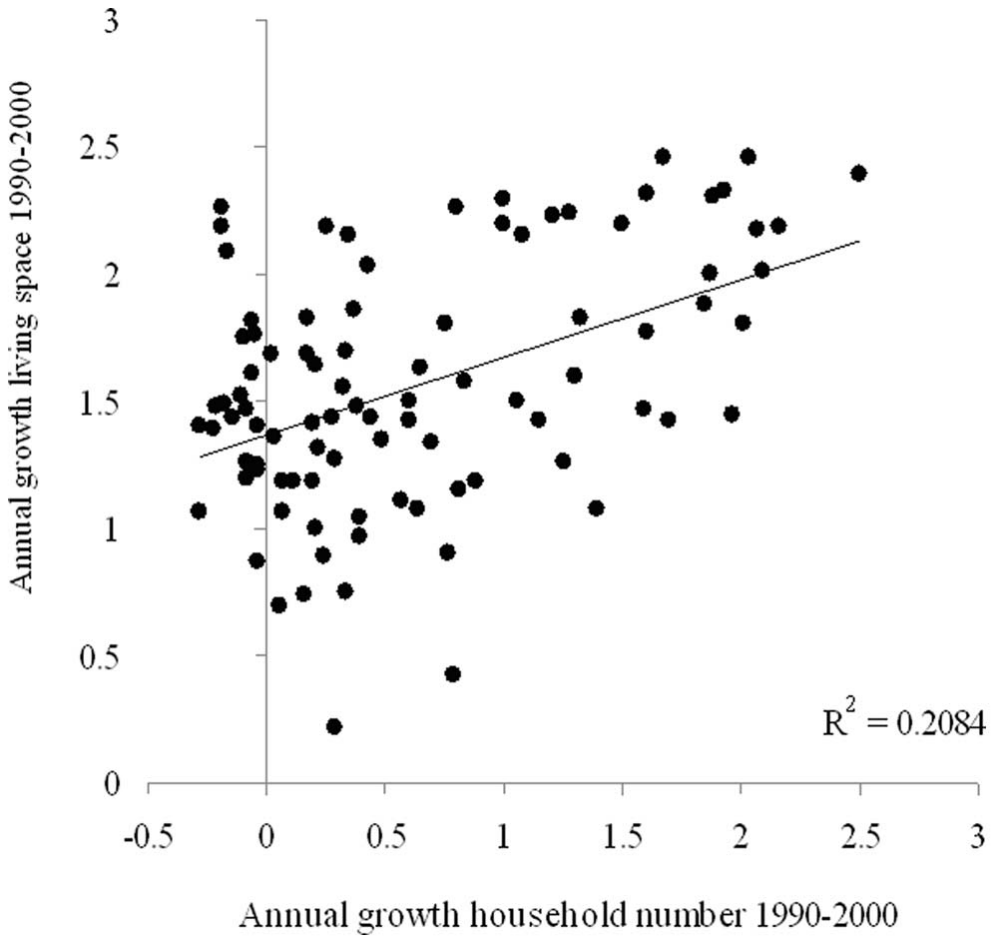
Linear regression showing the relationship between annual growth of household numbers and living space respectively for 1990–2000.

In addition to the decreasing household size and respective increase in household number being identified as driving forces behind the on-going growth of urban land area (our analysis and Liu et al., 2003∶532), there is another “invisible” variable that is responsible for urban land consumption under conditions of population decline, the per capita living space. Figure 4 illustrates that changes in per capita living space are correlated in a weakly positive way (R^2^ = 0.21 at p<0.001) with the growth rate of household numbers in large European cities. In accordance with similar findings by Kroll and Haase [27] for German agglomerations, our European sample shows that the growth rates of per capita living space remained positive, even when the household numbers began to decline (cf. again Fig. 1). This increase in per capita living space is certainly related to positive income development. In Europe, during the last few decades, the economy expanded in many ways. This first occurred in Southern Europe as a consequence of European enlargement until the 1980s, and after 1990, the expansion continued as a result of the post-socialist transition and EU integration. Household income increased almost everywhere, at least on average with the growing standard deviation, thereby allowing higher square footage or, to put it differently, for more per capita living space. Therefore, people can on average more easily afford to live in the small and smallest (1+2 person) households at the same square footage as 3+ households in the past [28]. Moreover, in cities with declining population numbers and a decreasing demand which is not automatically the case when a city loses population, land/housing costs less and prices/rents decrease. This effect makes it additionally easier to live in more space.

Finally, throughout Europe, the emergence of a modernist and more flexible lifestyle that includes a preference for more spacious and individual living promotes more living space or the deliberate choice to live alone [29], [30]. However, there is no automatism. Today, it is quite common for several small households to live together in one flat (flat shares), a phenomenon which is also quite common in large cities all over Europe [31], [32]. Finally, as a consequence of the current economic crisis, many young people may return to their parental homes or may not leave, which might also lead to a countertrend that would only become apparent in future statistical analyses.

In conclusion, small households, on average, consume more urban land area per capita than larger ones, and as our results show, small households, which are basically one-person households, have been growing in number from 1990 to 2006 throughout Europe, independent of whether population size or even total household number in a city grew or declined. In our argument, we reference Liu et al. (2003) but go beyond the argument presented there.

### The Urban Land Consumption Debate: no Simple Solution by Population or Household Number Decline but Form and Arrangements Matter

Is there a way to escape the negative consequences of further urban land area growth when neither a reduction in population nor household number works as a key to halt land consumption?

Obviously, the sustainable use of land is rather undermined by i) different forms or arrangements of households that greatly vary with respect to their demand for living space (indirectly: urban land) and related infrastructure but also may change for the same household over time due to changing preferences [26]; ii) the nature in which households are distributed over the territory of a city, i.e., whether a city is more compact or perforated, dense or sprawling (see here descriptions of sprawl and perforation in [33], [34], [35], [36] and iii) on household income, meaning that if greater affluence drives an increased ability to live alone, then affluence would also drive un-sustainability [37], [38].

Subsequently, one of the primary challenges facing cities and their planners in the future is to develop new urbanisation concepts that adapt the current shape and densities of cities to new household developments and the respective changes/needs in housing forms/arrangements, thereby finding a recipe to counteract the trend towards increasingly unsustainable land consumption. This approach can, according to our results, only be reached when the objective of limiting urban land consumption is linked to current and future housing arrangements [36]. This linking leads to a debate that has become an area of increasing focus in recent years, the threat of the compact city [33[, [39], [41], [42]. In particular, the objective of compactness has explicitly been related to questions such as: Under which conditions does compactness mean sustainable land and resource use [33], liveability for a variety of residential groups/household types/housing arrangements [34], or a just distribution of environmental goods and burdens [41]?

It is clear that there is no “one-policy-fits-all” approach to limit the “endless urban growth” in a sustainable way for the European realm [43]. There are, however, a number of examples and strategies to plan or create sustainable compactness, such as fostering lower-density or suburban-type housing in inner-city neighbourhoods [44], easing flat-share solutions for large flats, prioritising inward and infill developments (e.g., on urban brownfield sites) and supporting this approach by creating “housing moratoria” for suburban areas [45]. This approach would attract suburbanites to the inner sections of the city and enable the creation of a city that is denser, reflects a mixed-use area and has clear boundaries [36]. Compact cities are also favoured because urban land area can be reused, while rural land beyond the urban edge is protected [46].

Ultimately, we argue that good urban quality of life can be sustained, even with high concentrations of people, as urban re-densification fosters high accessibility to urban goods and services. Indeed, this approach allows the best connectivity between people, work and leisure, thereby minimising the amount of land needed per capita. This approach combines the prevailing concept of the compact city with the new demography and lifestyle-driven requirements of urban residents in the 21^st^ century, which include shorter commutes, good access to any type of infrastructure, and recreational green space that is “around the corner”. Recent research indicates that a number of European cities face trends toward reurbanisation, which are driven to a considerable extent by small(er) and young(er) residents/households [21], [47]. However, for Western countries, there has been an increasing number of retired households [48], [49] moving to inner-city areas, which could support urban planning to keep the city compact. Incorporating such new research findings into urban planning and design will lead to resolutions that counteract “endless urban growth”–a term coined by Burdett and Sudjic [50]–and would ideally create a win-win situation for land, resources and inhabitants in the “the finite city”.

### Conclusions

In this paper, we have shown that neither population decline nor the decrease in the total household number in cities lead to a decrease in land consumption in European cities. To the contrary, land consumption is assumed to be further increasing, even in cases in which household numbers decrease. We conclude that beyond population and (one-person) household number, there are variables such as living arrangements and types of housing, that seem to be more important for explaining the current growth of urban land area. In doing so, we clearly show that other variables must be explored to explain why land consumption and per capita living space expand regardless of population and household trends. Future research must consider other variables, such as changes in household types, age-group specific life styles and housing arrangements, and must also look at the spatiality of new land consumption in different types of cities to explain the direction and scope of resource consumption.

## Supporting Information

Table S1 **The list of cities and population number in 1990, 2000 and 2006.**
(DOC)
