# Supplementary material for: Endless Urban Growth? On the Mismatch of Population, Household and Urban Land Area Growth and Its Effects on the Urban Debate
Source: PLoS One. 2013 Jun 20;8(6):e66531. doi: 10.1371/journal.pone.0066531 (PMC3688765; doi:10.1371/journal.pone.0066531)
Supplement: Table S1 — The list of cities and population number in 1990, 2000 and 2006. (DOC) [file pone.0066531.s001.doc]

**Supplement Table S1** List of cities and population numbers in 1990, 2000 and 2006.

| **Country** | **City** | **population number** | | |
| --- | --- | --- | --- | --- |
|  |  | **1991** | **2001** | **2004** |
| Austria | Wien | 1539848 | 1550123 | 1598626 |
|  | Graz | 237810 | 226244 | 235477 |
|  | Linz | 203044 | 183504 | 185530 |
|  |  |  |  |  |
| Belgium | Brussels | 954045 | 978384 | 999899 |
|  | Antwerp | 467518 | 448709 | 455148 |
|  | Gent | 230246 | 226220 | 229344 |
|  | Charleroi | 206214 | 200578 | 200608 |
|  | Liège^b^ | 196303 | 351095 | 360361 |
|  | Bruges | 117063 | 116836 | 117025 |
|  |  |  |  |  |
| Bulgaria | Sofia | 1140795 | 1091772 | 1138950 |
|  | Varna | 316231 | 312889 | 312026 |
|  | Burgas | 211597 | 192390 | 189529 |
|  | Pleven | 137466 | 121880 | 115354 |
|  | Ruse | 190229 | 161453 | 158201 |
|  | Vidin | 68164 | 57395 | 53488 |
|  |  |  |  |  |
| Switzerland^a^ | Genève | 171042 | 177964 |  |
|  |  |  |  |  |
| Czech | Prague | 1214174 | 1169106 | 1170571 |
| Republic | Brno | 388296 | 376172 | 367729 |
|  | Ostrava | 327371 | 316744 | 311402 |
|  | Plzen | 173791 | 166118 | 162627 |
|  | Usti nad Labem | 98178 | 95436 | 93859 |
|  |  |  |  |  |
| Germany^b, c^ | Berlin | 3465748 | 3388434 | 3387828 |
|  | Hamburg | 1688785 | 1726363 | 1734830 |
|  | Munich | 1256638 | 1227958 | 1249176 |
|  | Cologne | 960631 | 967940 | 969709 |
|  | Frankfurt am Main | 663952 | 641076 | 646889 |
|  | Essen | 627269 | 591889 | 588084 |
|  | Leipzig | 542512 | 493052 | 498491 |
|  | Dresden | 502900 | 478631 | 487421 |
|  | Dortmund | 600669 | 589240 | 588680 |
|  | Düsseldorf | 578135 | 570765 | 572663 |
|  | Bremen | 554377 | 540950 | 545932 |
|  | Hannover | 523627 | 516415 | 515841 |
|  | Nurnberg | 500198 | 491307 | 495302 |
|  | Bochum | 400356 | 390087 | 388179 |
|  | Bielefeld | 324287 | 323373 | 328012 |
|  | Halle an der Saale | 299884 | 243045 | 238497 |
|  | Magdeburg | 274244 | 229755 | 226675 |
|  | Wiesbaden | 268069 | 271076 | 274076 |
|  | Göttingen | 128299 | 123822 | 122187 |
|  | Mülheim a.d.Ruhr | 176962 | 172332 | 170327 |
|  | Darmstadt | 141431 | 138457 | 140078 |
|  | Trier | 98958 | 100024 | 100163 |
|  | Freiburg im Breisgau | 195789 | 208294 | 213998 |
|  | Regensburg | 124398 | 127198 | 128917 |
|  | Frankfurt (Oder) | 84937 | 70308 | 65242 |
|  | Weimar | 62750 | 63522 | 64491 |
|  | Schwerin | 124084 | 99978 | 97110 |
|  | Erfurt | 218770 | 200126 | 202450 |
|  | Augsburg | 264852 | 257836 | 260407 |
|  | Bonn | 298227 | 306016 | 311938 |
|  | Karlsruhe | 279329 | 279578 | 284163 |
|  | Mönchengladbach | 265069 | 262963 | 261966 |
|  | Mainz | 179486 | 185293 | 186061 |
|  |  |  |  |  |
| Denmark | Copenhagen | 464773 | 499148 | 501664 |
|  | Aarhus | 264136 | 286668 | 293510 |
|  | Odense | 177639 | 183691 | 185206 |
|  | Aalborg | 155664 | 161661 | 163231 |
|  |  |  |  |  |
| Spain | Madrid | 3010492 | 2938723 | 3099834 |
|  | Barcelona | 1643542 | 1503884 | 1578546 |
|  | Valencia | 752909 | 738441 | 785732 |
|  | Seville | 683028 | 684633 | 704203 |
|  | Zaragoza | 594394 | 614905 | 638799 |
|  | Málaga | 522108 | 524414 | 547731 |
|  | Murcia | 328100 | 370745 | 398815 |
|  | Las Palmas | 354877 | 354863 | 376953 |
|  | Valladolid | 330700 | 316580 | 321713 |
|  | Palma de Mallorca | 296754 | 333801 | 368974 |
|  | Santiago de Compostela | 87807 | 90188 | 92298 |
|  | Vitoria/Gasteiz | 206116 | 216852 | 223702 |
|  | Oviedo | 196051 | 201154 | 209495 |
|  | Pamplona/Iruña | 180372 | 183964 | 191865 |
|  | Santander | 191079 | 180717 | 183799 |
|  | Toledo | 59802 | 68382 | 73485 |
|  | Badajoz | 122225 | 133519 | 139135 |
|  | Logroño | 122254 | 133058 | 141568 |
|  |  |  |  |  |
| France^a,c,d^ | Paris | 2152423 | 2125246 | 2181374 |
|  | Lyon | 1134687 | 1167532 | 1226249 |
|  | Toulouse | 521824 | 583229 | 651586 |
|  | Strasbourg | 423712 | 451240 | 467375 |
|  | Bordeaux | 624286 | 659998 | 702522 |
|  | Nantes | 505076 | 554478 | 579131 |
|  | Lille | 1067345 | 1091438 | 1107861 |
|  | Montpellier | 353184 | 412891 | 406139 |
|  | Saint-Etienne | 409836 | 384042 | 378753 |
|  | Le Havre | 260189 | 255082 | 246196 |
|  | Rennes | 326311 | 364652 | 386065 |
|  | Amiens | 166910 | 171240 | 173510 |
|  | Rouen | 386546 | 391375 | 392811 |
|  | Nancy | 256371 | 258268 | 258525 |
|  | Metz | 204967 | 213000 | 221623 |
|  | Reims | 207393 | 214448 | 211050 |
|  | Orléans | 245849 | 266446 | 272572 |
|  | Dijon | 231734 | 238309 | 243189 |
|  | Poitiers | 111381 | 123589 | 133755 |
|  | Clermont-Ferrand | 256132 | 260762 | 279621 |
|  | Caen | 205805 | 216181 | 218151 |
|  | Limoges | 179865 | 184241 | 190501 |
|  | Besançon | 161672 | 170696 | 175299 |
|  | Grenoble | 366682 | 374922 | 396657 |
|  | Ajaccio | 67686 | 63707 | 76331 |
|  | Tours | 248237 | 258170 | 264927 |
|  | Aix-en-Provence | 302133 | 332653 | 354660 |
|  | Marseille | 969323 | 981769 | 1023973 |
|  | Lens - Liévin | 251585 | 250199 | 246097 |
|  |  |  |  |  |
| Hungary^e^ | Budapest | 2016458 | 1777921 | 1697343 |
|  | Miskolc | 195433 | 184125 | 175701 |
|  | Nyiregyhaza | 115089 | 118795 | 116336 |
|  | Pecs | 170513 | 162498 | 156567 |
|  |  |  |  |  |
| Italy | Roma | 2775250 | 2546804 | 2542003 |
|  | Milano | 1369231 | 1256211 | 1271898 |
|  | Napoli | 1067365 | 1004500 | 1000449 |
|  | Torino | 962507 | 865263 | 867857 |
|  | Palermo | 698556 | 686722 | 679730 |
|  | Genova | 678771 | 610307 | 601338 |
|  | Firenze | 403294 | 356118 | 367259 |
|  | Bari | 342309 | 316532 | 314166 |
|  | Bologna | 404378 | 371217 | 373539 |
|  | Catania | 333075 | 313110 | 307774 |
|  | Venezia | 309422 | 271073 | 271663 |
|  | Verona | 255824 | 253208 | 258115 |
|  | Cremona | 74113 | 70887 | 71458 |
|  | Trento | 101545 | 104946 | 108577 |
|  | Trieste | 231100 | 211184 | 208309 |
|  | Perugia | 144732 | 149125 | 153857 |
|  | Ancona | 101285 | 100507 | 101545 |
|  | L'Aquila | 66813 | 68503 | 70664 |
|  | Pescara | 122236 | 116286 | 122083 |
|  | Campobasso | 50941 | 50762 | 51629 |
|  | Caserta | 69027 | 75208 | 78965 |
|  | Taranto | 232334 | 202033 | 199131 |
|  | Potenza | 65714 | 69060 | 68920 |
|  | Catanzaro | 96614 | 95251 | 94924 |
|  | Reggio di Calabria | 177580 | 180353 | 181440 |
|  | Sassari | 122339 | 120729 | 121849 |
|  | Cagliari | 204237 | 164249 | 162560 |
|  |  |  |  |  |
| Lithuania | Vilnius | 607346 | 554281 | 552800 |
|  |  |  |  |  |
| Luxembourg | Luxembourg | 75833 | 76688 | 83226 |
|  |  |  |  |  |
| Malta | Valletta | 359543 | 207155 | 209422 |
|  |  |  |  |  |
| Poland | Warszawa | 1655272 | 1688972 | 1692854 |
|  | Lodz | 854261 | 791727 | 774004 |
|  | Krakow | 746627 | 757942 | 757430 |
|  | Wroclaw | 638986 | 640804 | 636268 |
|  | Poznan | 587971 | 579343 | 570778 |
|  | Gdansk | 464308 | 461885 | 459072 |
|  | Szczecin | 410331 | 415748 | 411900 |
|  | Bydgoszcz | 377807 | 374352 | 368235 |
|  | Lublin | 339901 | 357156 | 355998 |
|  | Katowice | 367014 | 328103 | 319904 |
|  | Bialystok | 263884 | 290091 | 292150 |
|  | Kielce | 210699 | 212763 | 209455 |
|  | Torun | 199527 | 211269 | 208278 |
|  | Olsztyn | 158865 | 172652 | 173850 |
|  | Rzeszow | 148560 | 160431 | 159020 |
|  | Opole | 126404 | 130091 | 128864 |
|  | Gorzow Wielkopolski | 121549 | 125818 | 125578 |
|  | Zielona Gora | 111910 | 118268 | 118516 |
|  | Jelenia Gora | 92569 | 89560 | 87643 |
|  | Nowy Sacz | 75206 | 84453 | 84463 |
|  | Suwalki | 57627 | 68851 | 69113 |
|  | Konin | 78817 | 82500 | 81266 |
|  | Zory | 65424 | 63511 | 62964 |
|  |  |  |  |  |
| Portugal | Porto | 302472 | 263131 | 238954 |
|  | Braga | 141256 | 164192 | 170858 |
|  | Funchal | 115403 | 103961 | 100847 |
|  | Coimbra | 139052 | 148443 | 142408 |
|  | Setúbal | 103634 | 113934 | 120117 |
|  | Aveiro | 66444 | 73335 | 73626 |
|  |  |  |  |  |
| Romania | Bucuresti | 2107080 | 1936724 | 1927448 |
|  | Cluj-Napoca | 325090 | 299541 | 311528 |
|  | Timisoara | 339092 | 307786 | 303908 |
|  | Craiova | 306504 | 301364 | 299494 |
|  | Braila | 249662 | 223113 | 218984 |
|  | Oradea | 230276 | 209939 | 206463 |
|  | Bacau | 199623 | 185022 | 181126 |
|  | Arad | 198844 | 172759 | 169327 |
|  | Sibiu | 182923 | 156530 | 154543 |
|  | Targu Mures | 171211 | 151932 | 147734 |
|  | Piatra Neamt | 119628 | 113546 | 110288 |
|  | Calarasi | 75954 | 73763 | 73766 |
|  | Giurgiu | 71913 | 71227 | 70004 |
|  | Alba Iulia | 73196 | 67358 | 66293 |
|  |  |  |  |  |
| Slovenia | Ljubljana | 272650 | 270506 | 267563 |
|  | Maribor | 119828 | 114891 | 112558 |
|  |  |  |  |  |
| Slovakia | Bratislava | 442197 | 428672 | 425155 |
|  | Kosice | 235160 | 236093 | 235006 |
|  | Banska Bystrica | 83400 | 83056 | 81704 |
|  | Nitra | 84534 | 86726 | 85742 |
|  |  |  |  |  |

^a^ Reference year 1990 instead of 1991; ^b^ Reverence year 1992 instead of 1991; ^c^Reference year 1999; ^d^Reference year 2006 instead of 2004; ^e^Reference year 2005 instead of 2004
